# ICTV Virus Taxonomy Profile: Secoviridae 2022

**DOI:** 10.1099/jgv.0.001807

**Published:** 2022-12-02

**Authors:** Marc Fuchs, Jean-Michel Hily, Karel Petrzik, Hélène Sanfaçon, Jeremy R. Thompson, René van der Vlugt, Thierry Wetzel, ICTV Report Consortium

**Affiliations:** 1 School of Integrative Plant Science, Cornell University, Geneva, NY 14456, USA; 2 Institut Français de la Vigne et du Vin, 30240 Le Grau du Roi, France; 3 Institute of Plant Molecular Biology, 370 05 České Budějovice, Czech Republic; 4 Agriculture and Agri-Food Canada, Summerland, BC, Canada; 5 Ministry for Primary Industries, Auckland 1072, New Zealand; 6 Wageningen University and Research, Wageningen 6708 PB, Netherlands; 7 DLR Rheinpfalz, Neustadt an der Weinstrasse 67435, Germany

**Keywords:** ICTV report, *Secoviridae*, taxonomy

## Abstract

Members of the family *Secoviridae* are non-enveloped plant viruses with mono- or bipartite linear positive-sense ssRNA genomes with a combined genome of 9 to 13.7 kb and icosahedral particles 25–30 nm in diameter. They are related to picornaviruses and are members of the order *Picornavirales*. Genera in the family are distinguished by the host range, vector, genomic features and phylogeny of the member viruses. Most members infect dicotyledonous plants, and many cause serious disease epidemics. This is a summary of the International Committee on Taxonomy of Viruses (ICTV) report on the family *Secoviridae*, which is available at ictv.global/report/secoviridae.

## Virion

**Fig. 1.**
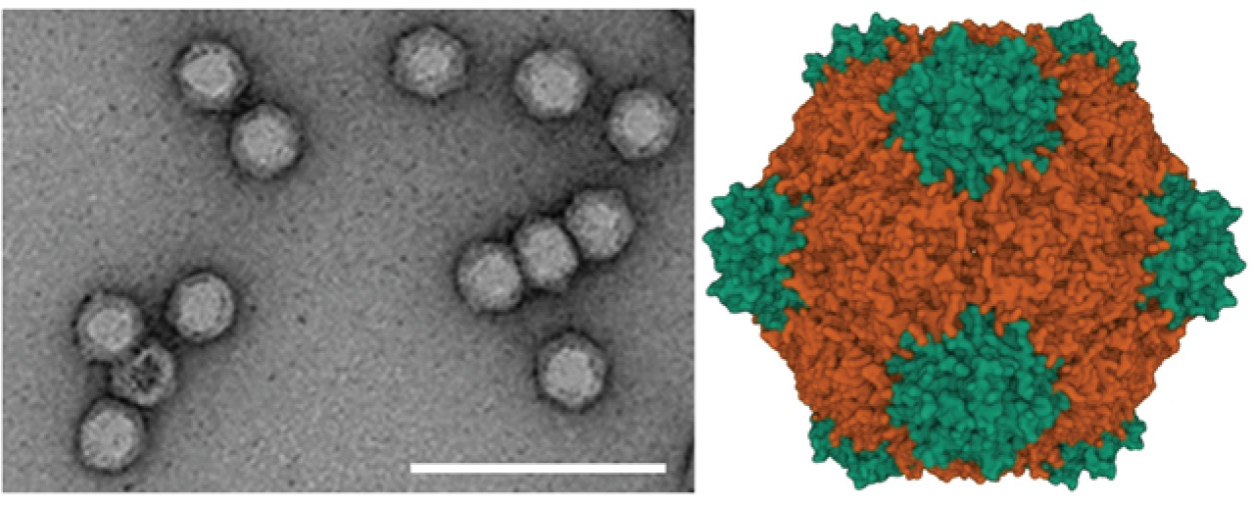
Virion structure of cowpea mosaic virus. Left: negative-contrast electron micrograph of purified particles. Bar: 100 nm. Note the empty particle with a stained interior (courtesy G.P. Lomonossoff and Springer Nature licence 5384990534567). Right: capsid crystal structure with the S (green) and L (orange) coat protein subunits forming three ß-sandwich domains (PDB,10.2210/pdb1NY7/pdb).

Virions are non-enveloped, 25–30 nm in diameter, with icosahedral symmetry (Table 1, Fig. 1). The two RNAs of viruses with a bipartite genome are encapsidated separately [1].

**Table 1.** Characteristics of members of the family *Secoviridae*

Example:	cowpea mosaic virus (RNA1, X00206; RNA2, X00729), species *Cowpea mosaic virus*, genus *Comovirus*
Virion	Non-enveloped, 25–30 nm in diameter with icosahedral symmetry
Genome	9.0–13.7 kb of positive-sense, mono- or bipartite RNA
Replication	Cytoplasmic in association with intracellular membranes derived from the endoplasmic reticulum
Translation	Directly from genomic RNAs as large polyproteins, which are cleaved by 3C-like proteinases
Host range	Plants (mainly dicots); transmission mainly by arthropods, nematodes, seeds or pollen
Taxonomy	Realm *Riboviria*, kingdom *Orthornavirae*, phylum *Pisuviricota*, class *Pisoniviricetes*, order *Picornavirales*; one subfamily with three genera, six additional genera, three subgenera and >100 species

## Genome

**Fig. 2.**
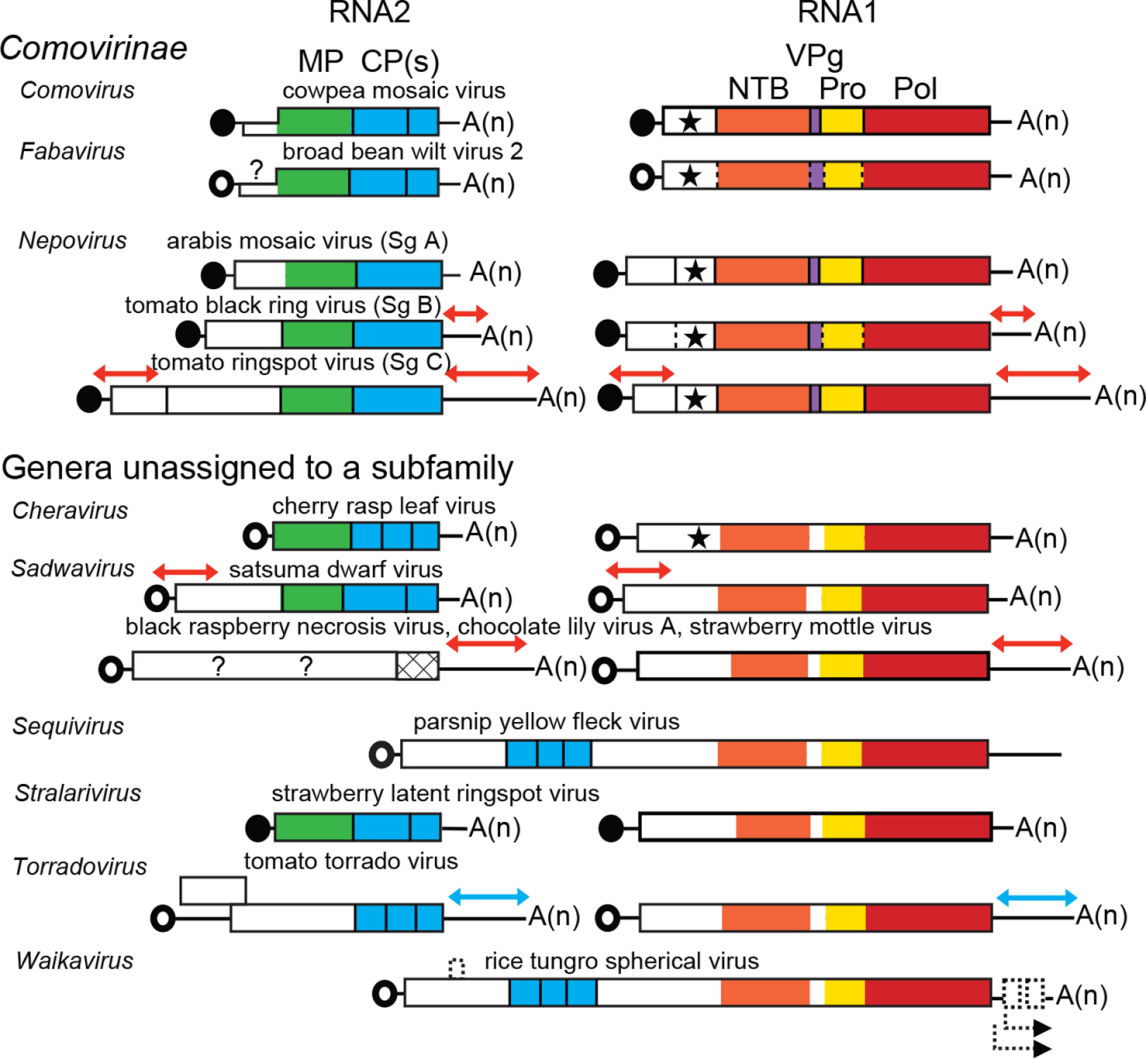
*Secoviridae* genome organization. Circles: experimentally confirmed (filled) or suspected (open) VPgs. A(n): poly (A) tails. Red and blue arrows: regions of extensive sequence identity between RNAs 1 and 2. ORFs (boxes) are shown with protein domains with conserved motifs for a movement protein (green), coat protein(s) (blue), RNA-directed RNA polymerase (Pol, red), 3C-like proteinase (3CL-Pro, yellow), VPg (purple), putative nucleoside triphosphate (NTP)-binding protein and helicase (orange), and protease cofactor (Co-Pro, star). Putative ORFs and subgenomic RNAs are depicted for waikaviruses with dotted rectangles and arrows, respectively. A glutamic proteinase (hatched) is shown for stramoviruses.

The genome consists of one or two molecules of linear positive-sense RNA covalently linked to a small protein (viral protein genome-linked, VPg) at the 5′-end and, usually, a 3′-terminal poly(A) tract [1]. Most RNAs encode a single polyprotein with a small additional partially overlapping ORF upstream in some RNA2 (Fig. 2).

## Replication

Viral proteins are expressed as large polyproteins, which are cleaved by virus-encoded 3C-like proteinases (3CL-Pros) and an additional glutamic proteinase for the RNA2 polyprotein of viruses in the subgenus *Stramovirus* (Fig. 2) [2]. The replication block contains the domain characteristics of NTP-binding proteins, 3CL-Pro and Pol. Replication occurs in association with intracellular membranes derived from the endoplasmic reticulum [1]. For viruses with a bipartite genome, neither RNA alone can systemically infect plants.

## Taxonomy

Current taxonomy: ictv.global/taxonomy. Viruses assigned to each genus are distinguished by host range, vector (beetles, aphids, longidorid nematodes, whiteflies, or leafhoppers) and genomic features [1,3,5]. Viruses in the same genus form monophyletic clades based on phylogenetic analyses of the coat protein(s) and the conserved Pro–Pol region (from the CG motif of the 3CL-Pro to the GDD motif of the Pol). Species demarcation criteria include <75 % amino acid sequence identity in the coat protein(s) sequences or <80 % amino acid sequence identity in the Pro–Pol region [1].

## Resources

Full ICTV Report on the family *Secoviridae*: ictv.global/report/secoviridae.

## Abbreviations

3CL-Pro: 3C-like proteinase
NTP: nucleoside triphosphate
Pol: RNA-directed RNA polymerase
VPg: viral protein genome-linked
